# Cryopreservation studies of an artificial co-culture between the cobalamin-requiring green alga *Lobomonas rostrata* and the bacterium *Mesorhizobium loti*

**DOI:** 10.1007/s10811-017-1270-8

**Published:** 2017-09-27

**Authors:** Christian J. A. Ridley, John G. Day, Alison G. Smith

**Affiliations:** 1 Department of Plant Sciences, University of Cambridge, Cambridge, CB2 3EA UK; 2 Scottish Association for Marine Science, Scottish Marine Institute, Oban, PA37 1QA UK

**Keywords:** Cryopreservation, Algal-bacterial co-cultures, Mutualism, Bacterial overgrowth, *Lobomonas rostrata*, *Mesorhizobium loti*

## Abstract

Algal-bacterial co-cultures, rather than cultures of algae alone, are regarded as having the potential to enhance productivity and stability in industrial algal cultivation. As with other inocula in biotechnology, to avoid loss of production strains, it is important to develop preservation methods for the long-term storage of these cultures, and one of the most commonly used approaches is cryopreservation. However, whilst there are many reports of cryopreserved xenic algal cultures, little work has been reported on the intentional preservation of both algae and beneficial bacteria in xenic cultures. Instead, studies have focused on the development of methods to conserve the algal strain(s) present, or to avoid overgrowth of bacteria in xenic isolates during the post-thaw recovery phase. Here, we have established a co-cryopreservation method for the long-term storage of both partners in a unialgal-bacterial co-culture. This is an artificial model mutualism between the alga *Lobomonas rostrata* and the bacterium *Mesorhizobium loti*, which provides vitamin B_12_ (cobalamin) to the alga in return for photosynthate. Using a Planer Kryo 360 controlled-rate cooler, post-thaw viability (PTV) values of 72% were obtained for the co-culture, compared to 91% for the axenic alga. The cultures were successfully revived after 6 months storage in liquid nitrogen, and continued to exhibit mutualism. Furthermore, the alga could be cryopreserved with non-symbiotic bacteria, without bacterial overgrowth occurring. It was also possible to use less controllable passive freezer chambers to cryopreserve the co-cultures, although the PTV was lower. Finally, we demonstrated that an optimised cryopreservation method may be used to prevent the overgrowth potential of non-symbiotic, adventitious bacteria in both axenic and co-cultures of *L. rostrata* after thawing.

## Introduction

Microbial consortia are ubiquitous across nature and are now being utilised in a wide variety of biotechnological applications, such as wastewater treatment (Unnithan et al. 2013). Consortia may be more productive than axenic cultures, due to effects such as resource-use efficiency and over-yielding (in which a community as a whole produces a greater yield than any single species in the community). Microbial consortia are successfully utilised in the hydrolytic and methanogenic bacterial communities of anaerobic digestion (Grosskopf and Soyer 2014), food production (Herve-Jimenez et al. 2009) and microbial fuel cells (Nishio et al. 2013), amongst other applications. Algal-bacterial consortia are being considered for application in advanced biorefineries, aquaculture and for environmental mitigation (Ramanan et al. 2016).

Algae possess great potential for industrial biotechnology as a result of their simple cultivation, minimal growth requirements and capability to be produced on non-arable land (Greenwell et al. 2010). However, mass microalgal culture remains a relatively expensive, energetic process and commercial success has largely been restricted to a few taxa for the production of high-value products (Varshney et al. 2015). High volume, low-value products, such as biofuels, have so far failed to be commercially viable, due in part to productivity losses at large scale by contamination of algal cultures by adventitious organisms, such as other algae, bacteria, fungi and zooplankton. Contamination is virtually impossible to avoid even in closed photobioreactors (Day et al. 2012). The fast growth rates of bacteria, in comparison to microalgae, and the abundance of nutrients in the culture medium, provides an ideal environment for bacterial proliferation (Sue et al. 2011). It is recognised that the contamination of algal cultures by bacterial pathogens is a significant contributor to the restricted growth of commercial-scale microalgal biotechnology for low-value products (Smith and Crews 2014). However, the presence of bacteria in algal cultures can be beneficial: mutualistic bacteria are extremely important to algal ecology and have been demonstrated to provide algae with vitamins (Croft et al. 2005; Paerl et al. 2015), iron (Amin et al. 2009) and phytohormones (Amin et al. 2015). This has led to the proposal that algal-bacterial co-cultures may be a mechanism to enhance the productivity of algal cultures, whilst reducing the likelihood of culture crash by adventitious or extraneous contaminating bacteria (Shurin et al. 2013; Kouzuma and Watanabe 2015).

In order to ensure the sustainability of production and the stability of algal-bacterial consortia, or microbial consortia more generally, it is necessary to develop preservation methods for their long-term storage. It is vital that biological inocula maintain genetic, phenotypic and functional stability so that biotechnological processes may be reproducible and consistent (Stacey and Day 2014). Whilst serial sub-culture is often the standard method used for algae (Harding et al. 2004), it may be unsuitable for the long-term storage of cultures, as genotypic and phenotypic drift can occur, with the potential loss of important characteristics of the strain (Day et al. 2005). Furthermore, culture maintenance by serial sub-culture can also result in contamination by adventitious bacteria and other microorganisms due to the increased likelihood of human error associated with frequent handling. For many microorganisms used in biotechnological applications, master stock cultures are stored as lyophilised (freeze-dried) or cryopreserved samples (Stacey and Day 2014). Unfortunately, lyophilisation of eukaryotic microalgae has proved largely unsuccessful, with little or no survival for most species (Day and Brand 2005). In contrast, cryopreservation (storage at an ultra-low temperature, between −80 and −196 °C) is a viable option for many algal taxa (Taylor and Fletcher 1998; Day and Brand 2005). Whilst the majority of methodology development has been performed on axenic cultures, many non-axenic taxa have also been successfully cryopreserved and examples can be seen on the websites of the major algal culture collections including CCAP (Culture Collection of Algae and Protozoa, www.ccap.ac.uk) and NCMA (National Center for Marine Algae and Microbiota, ncma.bigelow.org). Nonetheless, with the exception of an environmental consortium of mixed algae and bacteria, employed for bioremediation of a diluted effluent stream from an anaerobic digestion plant (Silkina et al. 2017), there is no report to date of a purposeful cryopreservation of a defined algal-bacterial co-culture.

In this study, we investigated whether a co-culture comprising the chlorophyte alga *Lobomonas rostrata* and the rhizobial bacterium *Mesorhizobium loti* could be successfully cryopreserved. This artificial system, first described by Kazamia et al. (2012), was set up as a model to study alga-bacterial mutualism at the molecular level, and is based on the fact that *L. rostrata* is dependent for growth on a supply of cobalamin (vitamin B_12_), which can be provided by *M. loti* in exchange for photosynthate. The interaction is characterised by a stable algal-bacterial ratio of about 30 *M. loti* cells per *L. rostrata* cell, and is highly regulated, with each partner’s growth dependent on that of the other species (Grant et al. 2014). We also determined whether *L. rostrata* could be cryopreserved alongside non-symbiotic bacteria without the occurrence of bacterial overgrowth upon thawing. Finally, a comparison of the practicality and applicability of standard equipment for the cryopreservation of unialgal-bacterial co-cultures was performed.

## Methods

### Cultivation methods

Axenic *Lobomonas rostrata* SAG 45–1 was cultured in autotrophic TP+ medium supplemented with vitamin B_12_ in the form of cyanocobalamin at a concentration of 100 ng L^−1^ as described by Kazamia et al. (2012). Cultures were maintained at 25 °C with 120-rpm shaking and illuminated by cool white fluorescent lamps with a photon flux density of 100 μmol photons m^−1^ s^−1^ in a 16:8 h light-dark regime. Stock cultures were maintained by transferring 1 mL of dense culture into 25 mL of fresh TP+ (+B_12_) once every 4 weeks. Axenicity of cultures was assessed by visual inspection of cultures grown on plates, prepared by serial dilution of 1 mL of culture on LB and TY agar plates following incubation for 3 days at 28 °C (Ridley 2016).

The sequenced strain of *M. loti* (MAFF303099) ( Kaneko et al. 2000) was cultured in TY broth (tryptone 5 g L^-1,^ yeast extract 3 g L^−1^, CaCl_2_.2H_2_O 0.875 g L^−1^) at 28 °C for 4 days. *Mesorhizobium loti* pre-culture aliquots (1 mL) were centrifuged and the biomass pellet was washed twice in sterile 1.5% NaCl solution to remove residual medium, before resuspension in 1 mL of the NaCl solution. Aliquots (1 mL) of dense *L. rostrata* culture were washed by centrifugation and resuspended in 1 mL sterile TP+ medium, and transferred into 25 mL of fresh TP+ without vitamin B_12_, to which 5 μL of the washed and resuspended *M. loti* culture was added. Co-cultures were maintained under the standard *L. rostrata* cultivation conditions for 7–10 days to allow the mutualism to establish and the ratio of *M. loti* to *L. rostrata* to reach between 10:1 and 30:1, which was confirmed by assessing cell densities by using a Beckman Coulter Z2 cell counter (UK), following the method developed for this co-culture by Kazamia et al. (2012). To produce artificially contaminated *L. rostrata* cultures, two bacteria were used, *Pseudomonas fluorescens* (MAFF76a) and *Curtobacterium flaccumfaciens*, both of which were derived from non-axenic *L. rostrata* cultures (Ridley 2016). An inoculum of 1 × 10^5^ cells mL^−1^ of each of these bacteria was transferred into *L. rostrata* and *L. rostrata* + *M. loti* cultures to reach a final *L. rostrata* to bacterial-contaminant ratio of 1:1. The *L. rostrata* / bacterial mix was then cryopreserved.

To test preservation of the mutualistic co-culture, thawed samples of cultures were used to inoculate TP+ medium and grown for several days under standard conditions. Cell counts of *L. rostrata* were determined with a Coulter counter, and those for *M. loti* by colony-forming units after plating serial dilutions on TY agar plates.

### Cryopreservation protocol

The majority of experiments performed in this study used a Planer Kryo 360 controlled-rate cooler (Planer plc, UK), to allow the precise control of parameters required to optimise the protocol. Samples with known numbers of algal cells from cultures of *L. rostrata* or *L. rostrata* + *M. loti* were pretreated before freezing by dilution 1:1 into TP+ medium (+B_12_ or −B_12_, respectively) containing different cryoprotectants/cryoprotective agents (CPA), namely dimethyl sulphoxide (DMSO), methanol (MeOH) or glycerol, at 5 or 10% (*v*/*v* in culture medium). After addition of the cryoprotectant, samples were incubated at room temperature for 10 min. Aliquots (1 mL) were then transferred to pre-labelled cryovials. A two-step cryopreservation protocol based on that established by Morris (1981), cooling at 1 °C minute^−1^ to −40 °C, with the addition of automated ice-nucleation at −5 °C was employed. After being held for 15 min at −40 °C, all samples were plunged into liquid nitrogen. Non-treated samples were simply diluted 1:1 into culture medium without CPA, and then frozen under identical conditions.

Comparative cryopreservation methods were performed using a Mr. Frosty passive cooler from Nalgene (Thermo Fisher Scientific Inc., USA) in place of the Planer Kryo 360 unit. The Mr. Frosty unit was prepared as per the manufacturers’ instructions, with the addition of 250 mL of isopropanol in the reservoir adjacent to the chamber containing cryogenic vials and was then placed in a refrigerator overnight to equilibrate at approximately 4 °C. The samples were placed into the Mr. Frosty unit and stored in a −80 °C freezer for 90 min to provide a nominal cooling rate of −1 °C minute^−1^ to −80 °C, before plunging into liquid nitrogen. All samples were transferred to a cryostorage dewar for storage in liquid phase liquid nitrogen for at least 1 week before thawing.

### Thawing and recovery procedure

Cryopreserved samples and non-cryopreserved controls were removed from liquid nitrogen and immediately placed into a 40 °C water bath until thawed. The exterior of the vials was then surface sterilised with 70% ethanol to minimise the risk of adventitious contamination. The samples were aseptically removed, followed by 10-fold dilution into sterile TP+ medium (+/− B_12_) to reduce final cryoprotectant concentration to < 0.5%. To test whether light-affected post-thaw viability (PTV) of cryopreserved samples, cells were either analysed immediately or incubated in the dark at 24 °C for 24 h prior to analysis.

### Assessment of post-thaw viability of *L. rostrata*

Post-thaw viability (PTV) of *L. rostrata* was calculated as the percentage of viable cells after cryopreservation versus the cell counts of the samples before cryopreservation. The viability of cells was assessed using the non-toxic fluorescent vital stain 6-carboxyfluorescein succinimidyl ester (CFSE) (Thermo Fisher Scientific Inc., USA), by addition of 0.4 μL of a 0.6 μM CFSE stock to 1 mL of culture, either before cryopreservation or after thawing a sample removed from liquid nitrogen. Samples were analysed by examination under epifluorescence at 400× magnification using mirror unit UMWSG2 (Olympus, Japan) and filter set 41020 (Chroma Technology Corp, USA). An additional confirmatory staining method using 3-amino-7-dimethylamino-2-methylphenazine hydrochloride (neutral red) from Sigma-Aldrich (USA) was used to assess cellular damage and viability after cryopreservation. In viable algal cells, vacuoles are stained red, whilst non-viable cells lack this localisation and display a yellow/orange cytosol (Zetsche and Meysman 2012). Cells were visualised on an Olympus BX51 epifluorescence microscope equipped with phase-contrast and DIC optics. At least 50 algal cells were counted for each sample. Data were analysed using Student’s *t* test or one-way ANOVA using Microsoft Excel. A *P* value of < 0.05 was considered statistically significant. All error bars display standard deviation from at least three replicate experimental runs.

## Results

### Cryopreservation of axenic L. rostrata and L. rostrata + *M. loti* co-culture

**Fig. 1:**
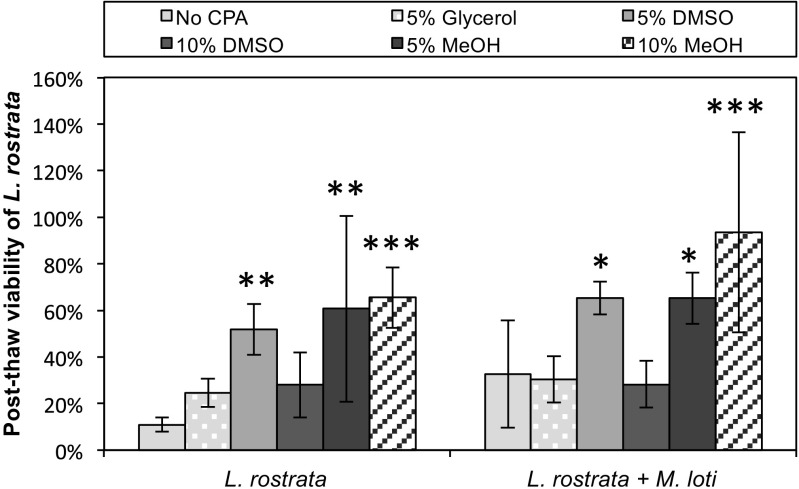
Effect of cryoprotective agents on post-thaw viability (PTV). Cultures of *L. rostrata* (left) and *L. rostrata + M. loti* (right) were cryopreserved with different CPAs, then thawed and tested for viability by CFSE staining. Values are mean of three samples ± standard deviation of the mean. Significance is marked by asterisks (*P < 0.05, **P < 0.01, ***P < 0.001)

**Fig. 2:**
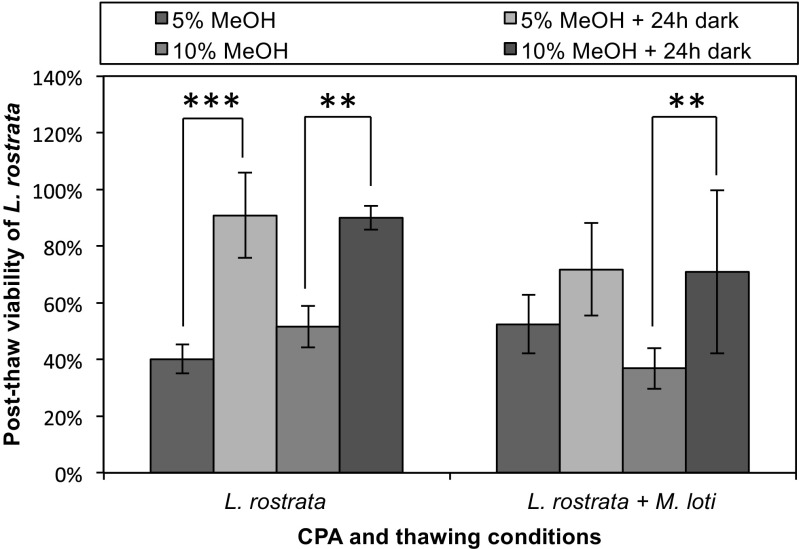
Effect of a dark incubation period on PTV. Cultures of *L. rostrata* (left) and *L. rostrata + M. loti* (right) were cryopreserved in 5 or 10% MeOH, and then either placed directly in the light after the removal from liquid N_2_, or incubated in the dark for 24 h after thawing before illumination. PTV was assessed as previously. Values are mean of three samples ± standard deviation of the mean. Significance is marked by asterisks, where *P < 0.05, **P < 0.01, ***P < 0.001

We first tested the survival of axenic cultures of *L. rostrata* and *M. loti* using different CPA, as shown in the left part of Fig. 1. Post-thaw viability was quantified by counting viable cells stained using CFSE as described in the Methods, and expressed as a percentage of cells originally cryopreserved. For the samples with no CPA, the PTV was 11 ± 3%. Whilst the use of 5% glycerol as the CPA resulted in an increased PTV (25 ± 14%), this was not significant (*P* = 0.1). The use of DMSO and MeOH as CPA all yielded higher PTV values than the absence of CPA, or 5% glycerol. The most effective CPA tested appeared to be 10% MeOH, resulting in a statistically significant increase in PTV of 65 ± 13% (*P* < 0.001). However, this value was statistically indistinguishable from 5% DMSO or 5% MeOH (52 ± 11% and 61 ± 40%, respectively). Previous cryopreservation studies have found that PTV can be enhanced by a period of dark incubation after thawing (Silkina et al. 2017). A 24-h dark incubation of axenic *L. rostrata* cells increased PTV significantly using both 5% (*P* = 0.0005) and 10% MeOH (*P* = 0.01) as CPA (Fig. 2).

**Fig. 3:**
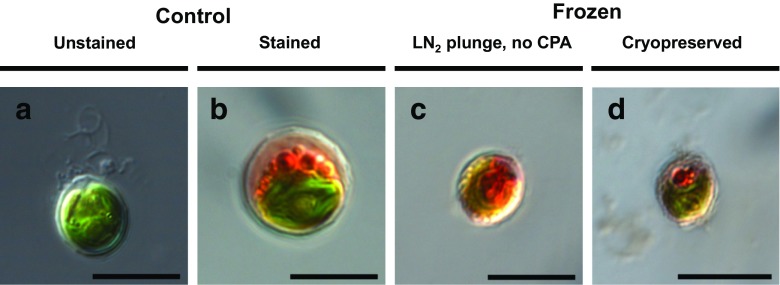
DIC microscopy of neutral red-stained *L. rostrata* cells. **a** Unstained, untreated *L. rostrata* cell. **b** Control, non-cryoprotected, non-cooled/frozen *L. rostrata* cell stained with neutral red. **c** Non-viable cell, post-thaw with damaged vacuolar membranes. **d** Viable neutral red-stained cells after cryopreservation employing *n* 5% MeOH as CPA, followed by thawing and 24-h dark incubation. Scale bar is 10 μm in length. Cell size is not indicative of a change in viability

Validation of the efficacy of the protocol was carried out with an additional vital stain using neutral red (Fig. 3). *Lobomonas rostrata* cells that had not been frozen contained multiple red-stained vacuoles indicative of viable cells (Fig. 3b). On subjecting cells to lethal cryo-stress by plunging samples directly into liquid nitrogen, staining indicates that the intracellular structures such as the cup-shaped chloroplast visible in Fig. 3a, were disrupted with the neutral red stain diffusely visible across the cell (Fig. 3c). In contrast, these features are retained in cells that had been cryopreserved using the standardised protocol (Fig. 3d).

After demonstrating successful cryopreservation of axenic *L. rostrata*, the protocol was then tested on the *L. rostrata* + *M. loti* co-culture. To ensure that cryoprotectant efficiency was consistent between axenic *L. rostrata* and the co-cultured algae and bacteria, the same CPAs were tested (Fig. 1, right-hand part). The optimal CPA was again found to be 10% MeOH (94 ± 43%), resulting in a significant increase in PTV compared to samples with no CPA (*P* < 0.001). There were no significant differences in PTV between axenic *L. rostrata* and the co-culture with *M. loti*. Again, a 24-h dark incubation period enhanced PTV compared to samples immediately transferred and incubated in the light (*P* = 0.004) as shown in Fig. 2 (right-hand side). Using this protocol, final post-thaw viability values of 91% (± 15%) for axenic *L. rostrata* and 72% (± 16%) for *L. rostrata* in co-culture with *M. loti* were obtained.

**Fig. 4:**
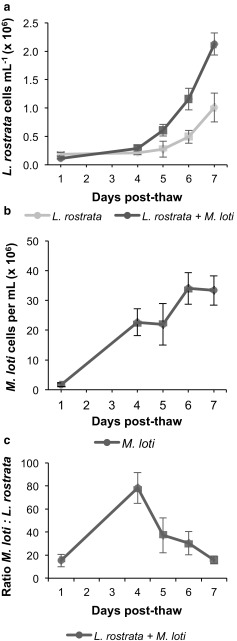
Regrowth of cultures after thawing from 6 months’ storage in liquid nitrogen. **a** Growth over 7 days of axenic *L. rostrata* (light grey) and *L. rostrata + M. loti* (dark grey) measured by cell counts in Coulter counter. **b** Growth over 7 days of *M. loti* in *L. rostrata + M. loti* co-culture, measured by CFUs. **c** Ratio of numbers of *M. loti: L. rostrata* cells. Values are mean of three samples ± standard deviation of the mean

To determine if the mutualistic bacterium *M. loti* survived the cryopreservation process, and to investigate the maintenance and stability of the symbiotic interaction after long-term storage in liquid nitrogen, cryopreserved co-cultures were thawed after ~ 6 month storage in liquid nitrogen and used to inoculate fresh TP+ medium. After 24-h dark incubation, cultures were placed in the light and, as shown in Fig. 4a, *L. rostrata* cells survived and regrew successfully, in fact more effectively than the axenic *L. rostrata* sample (light grey line). Similarly, *M. loti* cells from the co-culture grew well over the time course (Fig. 4b). Moreover, the co-culture medium contained no supplementation of vitamin B_12_ or a carbon source, whereas it was necessary to supplement the medium for the axenic cultures with vitamin B_12_. This indicates that the bacterium continued to supply this micronutrient to the alga, confirming that the mutualistic interaction had persisted. A further characteristic of this artificial co-culture is that there is regulation of the numbers of bacterial and algal cells, at an equilibrium ratio of ~ 10–30 to 1 (Kazamia et al. 2012; Grant et al. 2014). For the specific experiment shown here, the co-culture had an initial ratio of ~ 15 *M. loti* cells per *L. rostrata* cell, which rose to 78 (± 13) bacteria to algae 4 days after thawing, before dropping back to the original level (Fig. 4c).

### Cryopreservation of xenic algal cultures containing non-symbiotic bacteria

**Fig. 5:**
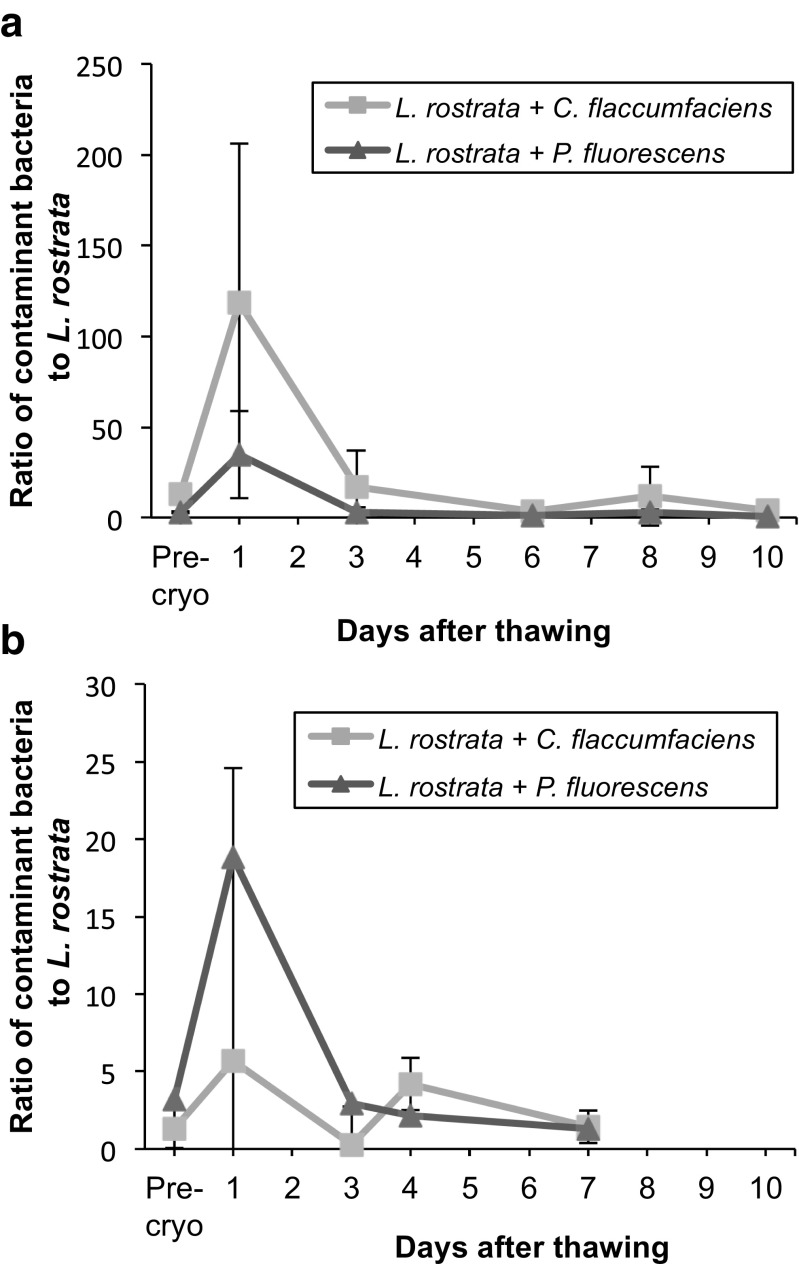
Testing for overgrowth by contaminating bacteria. Cultures of **a** *L. rostrata,* or **b** *L. rostrata + M. loti* were inoculated with either *P. fluorescens*, or *C. flaccumfaciens*, then cryopreserved and thawed as previously detailed using 5% MeOH as CPA and 24-h dark treatment. The number of contaminating bacteria was determined, and this is plotted as the ratio to *L. rostrata* cells. “Pre-cryo” is a control ratio calculated 1 day before cryopreservation took place. Values are mean of six samples ± standard deviation of the mean

We also wanted to determine whether *L. rostrata* could be effectively cryopreserved when non-mutualistic bacteria were present, whilst avoiding bacterial overgrowth upon thawing, and whether the presence of the mutualistic partner had any impact on this. Two bacteria, which had previously contaminated cultures of *L. rostrata* grown under outdoor conditions (Ridley 2016), were chosen as test species. *Pseudomonas fluorescens* (MFAF76a) is a suspected pathogen of *L. rostrata*, whilst *Curtobacterium flaccumfaciens* is a commensal bacterium with no effect on *L. rostrata* growth (Ridley 2016). To simulate contamination, an inoculum of 1 × 10^5^ cells mL^−1^ of each of these bacteria was added into cultures of either *L. rostrata* or *L. rostrata + M. loti* at a final *L. rostrata* to bacterial-contaminant ratio of 1:1. The *L. rostrata* / bacterial mix was then cryopreserved using the standardised protocol. After approximately 1 month storage under liquid nitrogen, samples were thawed and used to inoculate cultures as described above. Measurement of cell numbers (Fig. 5) revealed an initial increase in the ratio of the contaminating bacteria (*P. fluorescens* or *C. flaccumfaciens*) to *L. rostrata* in both axenic *L. rostrata* and *L. rostrata + M. loti* cultures in a similar pattern to that observed with *M. loti* (Fig. 4c). However, 3 days post-thaw, all ratios had reduced to be approximately equal to, or less than, pre-cryopreservation levels and no bacterial overgrowth was observed.

### Comparison of cooling rates

**Fig. 6:**
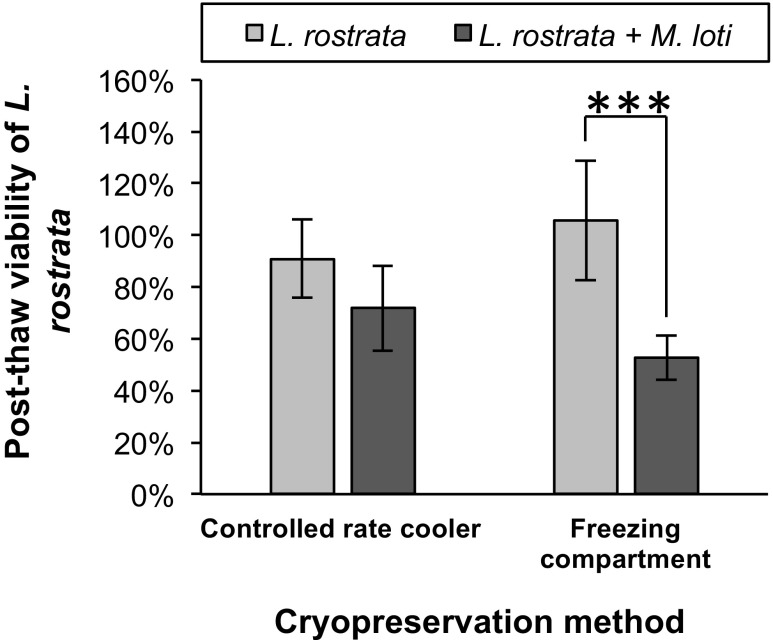
Comparison of different methods for cooling cryopreserved cultures. Cultures of either axenic *L. rostrata*, or *L. rostrata + M. loti* were cryopreserved using either a Planer Kryo 360 controlled-rate cooler or Mr. Frosty passive cooler, then thawed with 24-h dark treatment. PTV was assessed by CFSE staining. Values are mean of three samples ± standard deviation of the mean. Significance is marked by asterisks, where *P < 0.05, **P < 0.01, ***P < 0.001

A comparison was made between the Kryo 360 controlled-rate cooler method (used up to this point in the study) and a passive freezing compartment, Mr. Frosty, to assess whether this relatively low-tech approach could be employed for the cryopreservation of the microbial consortia studied. Using the Mr. Frosty passive cooler compartment was not found to have a significant difference on the PTV of axenic *L. rostrata* cultures, or on the *L. rostrate + M. loti* co-culture when compared between the two different freezing methods (Fig. 6). However, a significant reduction in PTV was observed for the *L. rostrata + M. loti* co-culture (52.6 ± 8.6%) compared to axenic *L. rostrata* (105.6 ± 23.1%) when using the Mr. Frosty (*P* = 0.001).

## Discussion

Whilst there have been previous studies that have investigated the implications of bacteria in the cryopreservation of algae (Amaral et al. 2013), and have cryopreserved communities of several different microbes (Kerckhof et al. 2014), to our knowledge, this study is the first to have successfully cryopreserved a mutualistic unialgal-bacterial co-culture with known characteristics (Kazamia et al. 2012) including a stable algal/bacteria ratio. A process of methodological refinement resulted in a highly efficient cryopreservation method that produced high post-thaw viability for axenic *L. rostrata* and *L. rostrata + M. loti* co-cultures. The optimal cryoprotectant tested for both *L. rostrata* and *L. rostrata + M. loti* was determined to be MeOH, in accordance with previous studies that identified MeOH as the more effective CPA for *Chlamydomonas reinhardtii* than DMSO, which is a more frequently employed cryoprotectant (Morris et al. 1979; Day and Brand 2005). Omission of a CPA, or the use of glycerol, was ineffective. The starting point for the development of this protocol was a two-step protocol developed for *C. reinhardtii*, which yielded PTV levels in our study of 72–91%, well above the minimum PTV threshold of 60% previously recommended by Day and Fleck (2015) to ensure the successful preservation of cultures. Moreover, the characteristics of the *L. rostrata* + *M. loti* co-cultures were recovered, even after 6-month storage in liquid nitrogen. Firstly, the nutrient exchange that is the basis of the mutualism was maintained since *L. rostrata* continued to grow without the need for vitamin B_12_ supplementation (Fig. 4a). Secondly, the ratio of *L. rostrata* cells to *M. loti* cells in an actively growing co-culture is stable at between 1:10 and 1:30 (Kazamia et al. 2012). In this study, the ratio was found to increase immediately after thawing, but decreased within expected values after several days (Fig. 4c). No overgrowth by *M. loti*, i.e. no bacterial “bloom” that results in restriction of growth or death of the algae, was observed, which suggests that co-cultures may be cryopreserved for at least several months. In theory, such stability might be maintained for decades, if not indefinitely (Grout 1995) and previous studies have demonstrated that a range of algal species remains stable after 20 years in cryostorage (Day et al. 1997). Additionally, we have demonstrated that algae may be cryopreserved, even with non-symbiotic bacteria, without the occurrence of bacterial overgrowth (Fig. 5). The issue of bacterial overgrowth in thawed algal cultures is a major concern in Biological Resource Centres (BRCs), as lysis of damaged cells releases additional bio-available nutrients into the recovery medium, which can stimulate a bacterial “bloom” and overgrowth of the algal culture. Heesch et al. (2012) determined that a minimum algal PTV of 25–50% was required to ensure rapid recovery of the macroalga *Ectocarpus*, and they included a wash step in the post-thaw process to remove the cryoprotectant and any carbon released from lysed cells. This approach has not been widely applied to microalgae as a centrifugation step is required, and this may result in additional damage to already compromised cells (Fleck 1998). In theory, antibiotics could be incorporated into the recovery medium, but in the case of CCAP this has not been routinely applied due to concerns of disrupting potential positive unialgal-bacterial interactions.

The enhanced PTV observed after a period of incubation in the dark following the thawing of cultures may be related to recovery from cryo-induced damage associated with the photosynthetic apparatus. Previous studies suggest that the inclusion of a short incubation period in the dark immediately after thawing cryopreserved cells could increase PTV levels (Day and Brand 2005) and further extension of this to at least 48 h resulted in PTV increasing from < 1 to > 75% for the benthic diatom *Planothidium frequentissimum* (Buhmann et al. 2013). Cryo-induced damage to photosynthetic apparatus in algae has also been observed in *Euglena gracilis* as a result of free radical production, due to metabolic uncoupling (Fleck et al. 2000). For some highly stress-tolerant algae like *Haematococcus pluvialis*, the presence of a coordinated antioxidant respond to oxidative stress, which is lacking in *E. gracilis*, may be the reason for its high cryo-tolerance (Fleck et al. 2003).

Choice of cryoprotectant may also influence the growth of bacteria post-thaw. The use of glycerol as the CPA to cryopreserve the marine red alga *Gracilaria tikvahiae* has been reported to be associated with bacterial overgrowth and subsequent culture contamination (van der Meer and Simpson 1984). Amaral et al. (2013) explored the implications of cryoprotectant choice on both efficacy of the cryopreservation procedure and the implications to the proliferation of partner organisms in non-axenic, mucilaginous algae and noted an increase in bacterial overgrowth when methanol, versus DMSO, was used as the CPA for a variety of microalgal species. However, in the present study, the use of methanol as CPA was not associated with bacterial overgrowth. Amaral et al. (2013) recovered, on average, slightly less than 50% of the cells after cryopreservation using DMSO or MeOH as CPA, whilst in this study, up to 71% PTV was achieved in the best case. Therefore, it is possible that the high PTV we achieved prevented the overgrowth of the commensal bacteria.

A final component of this study was to determine whether a specialist controlled-rate cooler (Planer Kryo 360) was necessary for the cryopreservation of unialgal-bacterial co-cultures, or if a passive freezer compartment would be sufficient. No significant difference was observed between the PTV of *L. rostrata* and *L. rostrata + M. loti* using the controlled-rate cooler. Similarly, no significant differences were detected for axenic *L. rostrata* or the co-culture in the Planer compared to the Mr. Frosty. However, a significant decrease in PTV was identified for the co-culture when using the Mr. Frosty passive freezer compartment, compared to axenic *L. rostrata*. This result suggests that for axenic algal strains, the quick and easy Mr. Frosty method may be sufficient, but for the cryopreservation of unialgal-bacterial co-cultures, a controlled-rate cooler may be necessary. The reasons for such a difference are unclear, but more precise control of the cooling rate and the possibility of controlling the point at which ice-nucleation/seeding occurs may be factors. It is possible that the two organisms differentially cryo-dehydrated in Mr. Frosty, due to the length of the treatment and the fact that the controlled-rate cooler did not go below −40 °C, whereas the Mr. Frosty was in a −80 °C freezer. During this phase of the cryopreservation process, cells are trapped in brine channels, which in sensitive taxa results in both physical damage and osmotic stress (Day and Fleck 2015). It is conceivable that the damage resulting from these differential stresses somehow disrupted the mutualism in the co-culture, perhaps triggering the observed overgrowth of *M. loti* upon the lysis of *L. rostrata* cells using this cooling method.

The techniques presented in this study demonstrate the ability to cryopreserve a unialgal-bacterial co-culture. Using a well-characterised model system, we demonstrated that the mutualism, evidenced by the algal/bacteria ratio observed, remained stable after the cryopreservation process and this indicates the possibility of successfully cryopreserving more complex microbial consortia. There are many examples of algal-bacterial consortia in development that may be applied to industrial biotechnology in the near future (Do Nascimento et al. 2013; Kouzuma and Watanabe 2015; Cho et al. 2015), which may benefit from such a technique. We believe that cryopreservation is the most suitable method to ensure the availability of stable and functional inocula for industrial biotechnology, particularly if complex consortia are to be utilised.
